# A randomized study on the effect of a wearable device using 0.75 Hz transcranial electrical stimulation on sleep onset insomnia

**DOI:** 10.3389/fnins.2024.1427462

**Published:** 2024-10-23

**Authors:** Stephen B. Simons, Maria Provo, Alexandra Yanoschak, Calvin Schmidt, Isabel Gerrard, Michael Weisend, Craig Anderson, Renee Shimizu, Patrick M. Connolly

**Affiliations:** Intelligent Systems Laboratory, Teledyne Scientific & Imaging, Durham, NC, United States

**Keywords:** sleep onset, insomnia, wearables, transcranial electrical stimulation, tDCS, anxiety, EEG

## Abstract

**Introduction:**

The normal transition to sleep is characterized by a reduction in higher frequency activity and an increase in lower frequency activity in frontal brain regions. In sleep onset insomnia these changes in activity are weaker and may prolong the transition to sleep.

**Methods:**

Using a wearable device, we compared 30min of short duration repetitive transcranial electric stimulation (SDR-tES) at 0.75Hz, prior to going to bed, with an active control at 25Hz in the same individuals.

**Results:**

Treatment with 0.75Hz significantly reduced sleep onset latency (SOL) by 53% when compared with pre-treatment baselines and was also significantly more effective than stimulation with 25Hz which reduced SOL by 30%. Reductions in SOL with 25Hz stimulation displayed order effects suggesting the possibility of placebo. No order effects were observed with 0.75Hz stimulation. The decrease in SOL with 0.75Hz treatment was proportional to an individual’s baseline wherein those suffering from the longest pre-treated SOLs realized the greatest benefits. Changes in SOL were correlated with left/right frontal EEG signal coherence around the stimulation frequency, providing a possible mechanism and target for more focused treatment. Stimulation at both frequencies also decreased perceptions of insomnia symptoms measured with the Insomnia Severity Index, and comorbid anxiety measured with the State Trait Anxiety Index.

**Discussion:**

Our study identifies a new potential treatment for sleep onset insomnia that is comparably effective to current state-of-practice options including pharmacotherapy and cognitive behavioral therapy and is safe, effective, and can be delivered in the home.

## 1 Introduction

Insomnia is the most common sleep disorder with estimates of over 30% of adults reporting symptoms in a given year, and greater than 20% of the population meeting the criteria for a formal diagnosis (Bjorøy et al., 2020; Cénat et al., 2021; Dopheide, 2020; Morin et al., 2006; Roth, 2007). Due to its prevalence, it carries an annual loss of quality-adjusted life years greater than many other medical and psychiatric conditions and is estimated to cost the United States more than $100 billion per year (Olfson et al., 2018; Wickwire et al., 2016).

Insomnia symptoms generally fall into one of two categories involving issues with either sleep onset (difficulty falling asleep) or sleep maintenance (difficulty staying asleep). An investigation into the prevalence of different subtypes suggests that more than 80% of individuals with insomnia suffer from difficulties with sleep onset making it the most commonly reported issue (Bjorøy et al., 2020). Despite the scale of the problem, current therapeutic options suffer from significant limitations.

Over the counter remedies including melatonin, nutritional supplements and behavioral interventions such as meditation/relaxation have limited efficacy, particularly in those suffering from chronic insomnia (Choi et al., 2022). Pharmacotherapy, including the widely prescribed z-class of drugs (e.g., zolpidem), have demonstrated efficacy in improving sleep outcomes but come with risk of negative side effects, habituation and withdrawal (Edinoff et al., 2021). Cognitive behavioral therapy (CBT) is currently the most effective therapy for insomnia, but suffers from limited access due to the general lack of licensed professionals (Thomas et al., 2016). Patient follow through is also an issue with CBT due to the need for several weeks of treatment prior to efficacy (Koffel et al., 2018).

Recently, neuromodulatory technologies such as transcranial magnetic stimulation (TMS), or transcranial electrical stimulation (tES) have been investigated for their ability to treat insomnia. These technologies are appealing since they are applied noninvasively, have limited side effects and lack the possible risks of addiction and withdrawal that come with pharmacotherapy. Studies using TMS have shown some efficacy to improve sleep outcomes, but enthusiasm for this approach is dampened by the need to receive treatments in the clinic. tES by comparison, can easily be delivered in the home and is less costly than TMS, but has not yet demonstrated efficacy to treat insomnia (Krone et al., 2023).

The purpose of this study was to investigate the efficacy of 0.75 Hz tES to reduce the time to sleep onset, improve overall sleep quality and reduce the subjective symptoms of sleep onset insomnia. In this study we used a recently developed wearable neurotechnology which delivers programmable tES to frontal brain regions to test the efficacy of this treatment in the home.

## 2 Materials and methods

Our study investigated the efficacy of 0.75 Hz tES delivered with a wearable device to improve sleep onset. Efficacy was compared with pre-treatment baselines and an active control using 25 Hz stimulation with the same device in the same subjects.

### 2.1 Study design

Our study design was a randomized within-subjects, patient-blind, crossover trial comparing treatment with 150-500 μA, 0.75 Hz SDR-tES with an active control arm of 100 mA, 25 Hz SDR-tES in a population of otherwise healthy adults with sleep onset insomnia.

#### 2.1.1 Participants

This study tested the hypothesis that electrical stimulation can improve sleep onset insomnia. Inclusion criteria for the study required subjects to be between 21 and 70 years old and have an Insomnia Severity Index (ISI) score of ≥8 indicating a likelihood of at least subthreshold insomnia (ISI survey given during screening). Baseline monitoring with actigraphy was used to confirm the presence of long sleep onset latency consistent with insomnia. Criteria for participation with the wearable device were defined prior to study start and required participants to have an average baseline sleep onset latency of >30 min with at least 3 out of 7 nights also >30 min consistent with criteria defined by the American Academy of Sleep Medicine and the Diagnostic and Statistical Manual of Mental Disorders, 5th edition (DSM-V-TR).

Subjects with implanted metal in the body, or a history of epileptic seizures were excluded due to additional risk from electrical stimulation. Those with diagnosed sleep disorders other than insomnia were also excluded. None of our subjects used prescription or over-the-counter medications for sleep or sedating drugs (e.g., antihistamines) during a period of at least 2 weeks prior to enrollment and through completion of their time in the study. Our study population demographics are shown in Table 1.

**Table 1:** Participant population demographics.

	0.75 Hz 1st	25 Hz 1st
Gender		
Female	8	6
Male	4	6
Age		
Median	52	50.5
Range	26–67	30–63
21–32	1	2
33–45	3	3
46–58	5	6
59–70	3	1
ISI		
Mean	14.5	15.75

#### 2.1.2 Randomization sequence and blinding

Participants were block randomized for order to either the 0.75 Hz treatment condition or the 25 Hz active control and were stratified according to baseline ISI scores. Block size of 4 participants was selected and was generated at random prior to participant enrollment. All participants were enrolled and assigned to the appropriate treatment arms by the study coordinator. Participants were blind to their order of treatment. It was not possible to blind the research coordinators due to a need to quality control each wearable device during their use on the study. This required visual inspection of the voltage trace which reveals the frequency/amplitude of the stimulation.

#### 2.1.3 Sample size

Target sample size of 24 patients was determined using power analysis based on results in an earlier pilot cohort investigating the primary effect of 0.75 Hz stimulation on SOL (*d*’ = 1.14). A total of 45 participants were enrolled over the course of this study with 24 participants completing all study procedures. 3 participants were dismissed for failure to adhere to study procedures and 18 participants did not participate in sessions with the wearable device because they did not meet the inclusion criteria of exhibiting evidence of insomnia during baseline testing.

### 2.2 Wearable device

**Figure 1:**
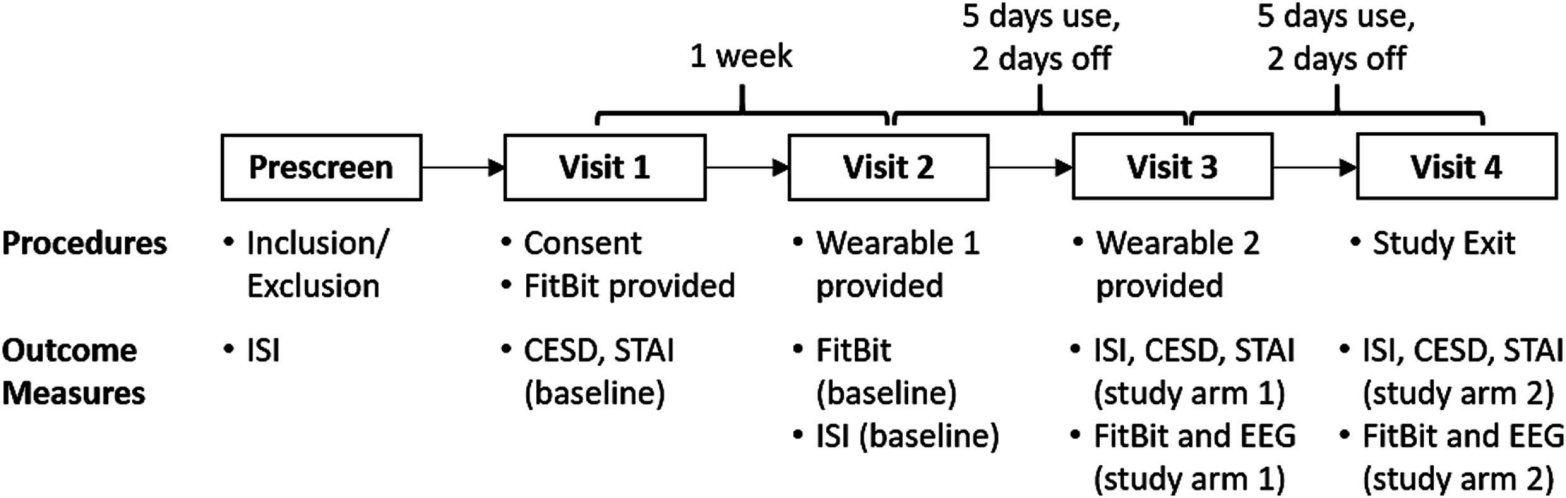
Within-subjects, active-control crossover design. Our translational study observed sleep at home using a wrist worn FitBit. Our SDR-tES pre-sleep interventions were tested over 5 days using our wearable neurotechnology, with a 2-day washout period. Both treatment conditions (0.75 Hz treatment or 25 Hz sham) were tested in each subject and the order was counterbalanced across subjects. Each participant made four visits to our laboratory for data and equipment exchange. FitBit sleep data, clinical questionnaires (ISI, CESD, STAI), and EEG data were collected during lab visits according to the schedule shown and compared against pre-treatment baselines and across treatment arms.

We used a wearable device developed by Teledyne Scientific for at home brain sensing and delivery of tES interventions. The device is shown in Figure 1 and delivers a previously described protocol for SDR-tES through two pairs of stimulating electrodes (Cellini et al., 2019). The anodes are located proximal to Fp1/Fp2 and the cathodes are placed on each corresponding mastoid. The device also contains three EEG sensing channels located proximal to FpZ, AF7, and AF8. EEG sensors were a commercially available dry electrode (Research, 2013). The device communicates wirelessly via Bluetooth low energy to a mobile phone which controls the device through a user-friendly application. The application performs a guided walkthrough on the setup of the device and performs a number of functional and safety checks such as ensuring good electrode connectivity determined by skin impedance. Once all safety checks are complete, the device collects 60s of EEG baseline data prior to initiating the stimulation protocol. The wearable device also logs the timing of each stimulation train along with start and end times of each session enabling comparison to sleep behavior data and ensuring that intervention protocols are successfully delivered. All EEG data and system logs are saved to the paired mobile device and were downloaded during participant visits to the lab for offline analysis.

#### 2.2.1 Interventions

Our device applies oscillating direct current bilaterally through each pair of hydrogel stimulating electrodes (Little PALS, Axelgaard) (Axelgaard, 2022). Finite element modelling suggests that this montage distributes current to frontal cortices (see Figure 1) (Thielscher et al., 2015). The oscillating current is applied with a trapezoidal waveform consisting of 4 segments of equal duration (0-current, rising phase, peak current, falling phase). Trains of stimulation are applied for 8 s and then turned off for 10 s. This pattern is delivered 100 times over a 30-min session. In the 0.75 Hz treatment condition, the waveform is applied at peak amplitudes ranging between 150 and 500 μA, while in the 25 Hz active control condition it is applied consistently at 100 μA. In the treatment condition, peak stimulation amplitude is scaled according to the measured skin impedance such that: at impedances >80kOhms, peak current is 150 μA, for impedances >50kOhms and < =80kOhms, peak current is 260 μA, and for impedances <=50kOhms peak current is 500 μA. In typical cases, skin impedance may exceed 100kOhms at the start of a session and gradually decrease following stimulation, to stable minimums between 20-50kOhms. The peak current amplitude in the 0.75 Hz treatment condition was varied to minimize discomfort from the stimulation that could otherwise occur at high skin impedances.

### 2.3 Study procedures

**Figure 2:**
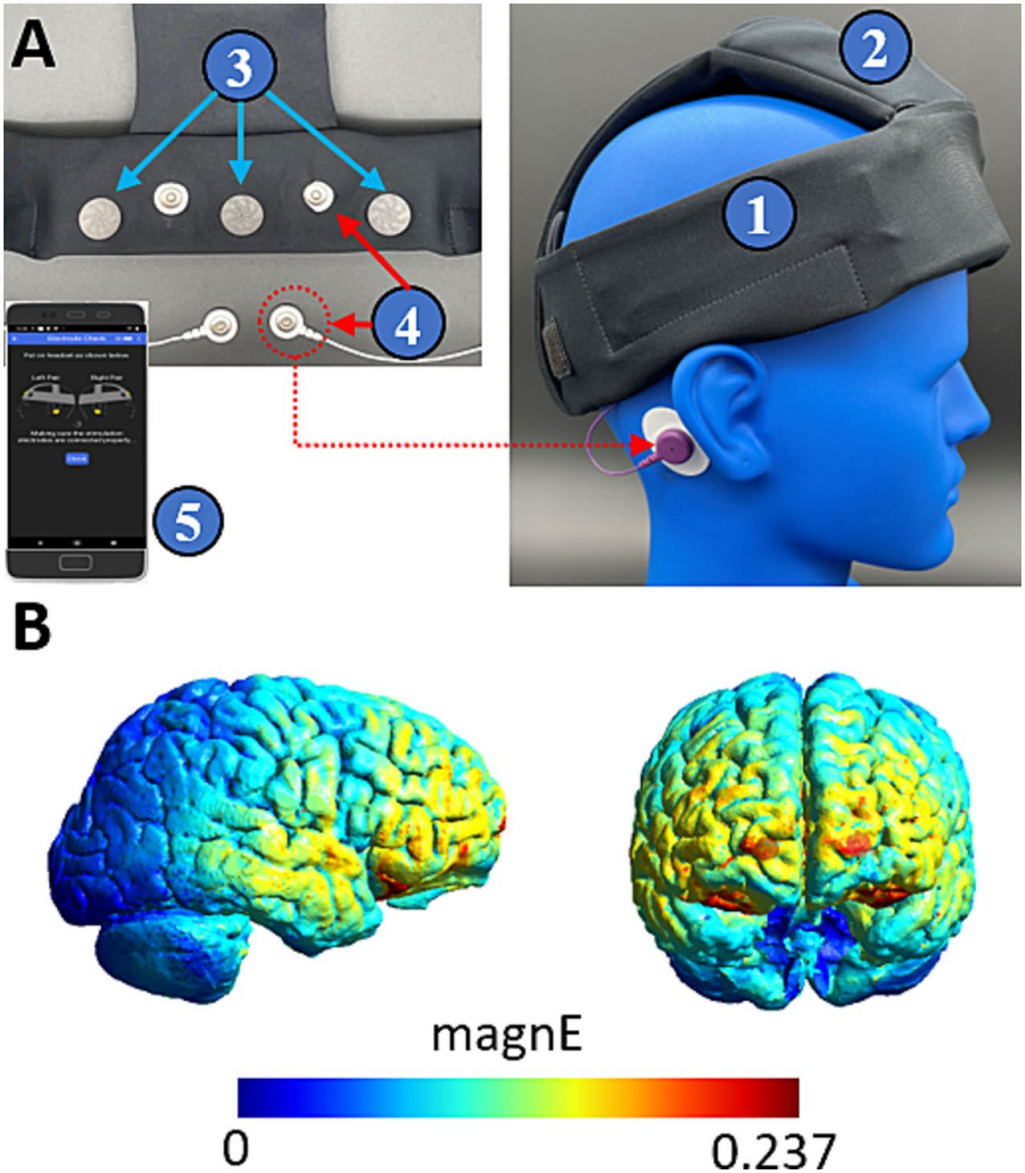
Teledyne’s wearable neurotechnology delivers transcranial electrical stimulation to frontal cortices. (**A**) The device is a wearable headband (1) with onboard electronics (2) and contains three EEG sensing channels (3) and two pairs of stimulating channels (4). The device is controlled by a mobile phone application (5) which provides a guided walkthrough on setup and delivery of the intervention. (**B**) Finite element modelling shows that stimulation delivers widespread current to frontal cortices. Color coding indicates the magnitude of the electric field driven with a 260 μA peak current.

This study was reviewed and approved by a private institutional review board at Western Copernicus Group (WCG) prior to enrollment of human subjects. Our study protocol is shown in Figure 2. Each participant spent 3 weeks enrolled in the study and made four visits to our laboratory. We used the FitBit Inspire 3 health tracker to track the sleep of participants at home across the entire 3 weeks. Baseline sleep patterns were captured and quantified during the first week. Each participant manually recorded the time that they laid down with the intent to fall asleep each night (“lights out”) in a written sleep diary. Sleep onset was defined as the start of the first continuous 10-min window of sleep measured with the FitBit from the recorded “lights out” time. During the second and third weeks, participants were also sent home with a Teledyne sleep wearable device (headset and accompanying mobile phone) and asked to wear the device for the first 5 nights of the week. Participants were instructed to wear the device for the 31-min intervention time, then remove the device, record their “lights out” time, and attempt to go to sleep. On nights 6 and 7 they were asked not to wear the headset to washout any residual effects from stimulation. One-way ANOVA tests revealed no significant differences between baseline and washout intervals for any dependent variable and are not reported. Each participant received 0.75 Hz on 1 week and 25 Hz on another week during the two-week intervention period. The order of the 0.75 Hz and 25 Hz interventions was randomized.

#### 2.3.1 Data collection

Three types of human subjects data were collected in this study: sleep behavior data, subjective clinical questionnaires, and electroencephalography (EEG) data collected by our wearable device. Sleep behaviors including: sleep onset latency, total sleep time and wake after sleep onset (WASO) were captured using the FitBit Inspire 3. Participants were also asked to keep a written record of “lights out” time indicating when they laid down with the intent to fall asleep. Subjective clinical questionnaires for insomnia, anxiety and depression were collected during each visit to the laboratory. We used the Insomnia Severity Index (ISI) to assess insomnia symptoms. The ISI is a 7-question screening tool that assesses a person’s satisfaction with their sleep and the degree to which insomnia interferes with daily functioning on a 7-point Likert scale. It is a widely used clinical instrument for the identification and severity assessment of insomnia. We used the State Trait Anxiety Index (STAI, State sub-form) to assess anxiety. The state sub-form is a 20-question instrument used to assess acute anxiety and to distinguish it from depressive symptoms. All items are rated on a 4-point scale from “Almost Never” to “Almost Always.”

We used the Center for Epidemiological Studies Depression scale (CES-D) to assess depression. The CES-D is a 20-question inventory that assesses how often symptoms associated with depression have been experienced in the past week. It is rated on a 0–3 scale with 0 = “Rarely or None of the Time” and 3 = “Most or Almost All the Time.”

#### 2.3.2 Outcome measures

Our primary outcome measure is SOL. Secondary outcome measures included all other sleep behavior variables as well as the subjective clinical questionnaires (ISI, STAI) defined above. Sleep behaviors were assessed each night and clinical questionnaires were assessed once per week, during each visit to our laboratory.

#### 2.3.3 Statistical methods

Statistical testing was performed in MATLAB. No significant difference was observed between baselines of either treatment-order group (Wilcoxon Rank Sum Test) enabling baseline norming to eliminate this source of variance from consideration. Comparison between changes in each dependent variable (i.e., SOL, time asleep) were baseline normed and tested using a 3-factor, repeated measures analysis of variance test using within subjects factors of Condition (0.75 vs. 25 Hz), and Day of use and a between subjects factor of Group (order of treatment). Missing data was imputed using random substitution from the entire pool of data for that variable (*n* ~ 240 samples = 5 days x 24 participants x 2 treatment conditions). The amount of missing data was ~8% for 0.75 Hz and ~ 6% for 25 Hz. To eliminate possible random impacts to the statistics from the imputation, ANOVA testing was performed with a Monte Carlo simulation with 10 repetitions and the resulting F-stat values were averaged. Across factors group means interactions were investigated using multiple comparisons testing.

Statistical testing between baseline and each treatment condition was done by averaging each participant’s data for each block and then performing a paired *t*-test. Statistical significance was corrected for multiple comparisons across treatment arms using the Bonferroni method.

All EEG data analysis was carried out using scripts written in MATLAB. Each recorded session with our wearable device contains 60 s of baseline EEG prior to stimulation. Baseline EEG and response data after each stimulus train were segmented into 4 s epochs for the purpose of artifact rejection and spectral processing. A segment was rejected if its raw voltage trace exceeded +/− 300 μV indicating non-physiological noise, or if the signal variance was <25 μV indicating a poor connection of the sensor channel to the skin. Raw signals were bandpass filtered from 0.5 to 50 Hz. Spectral analysis and magnitude squared coherence were performed using standard MATLAB functions for signal processing.

## 3 Results

### 3.1 Treatment with 0.75 Hz SDR-tES reduces sleep onset latency

The actigraphy data, measured with FitBit, included 152 baseline nights, 112 nights with 0.75 Hz stimulation, and 113 nights with 25 Hz stimulation. Table 2 describes the average sleep characteristics across participants (*N* = 24) during baseline, and both study arms. Results of a 3-factor, repeated measures ANOVA test indicated a significant effect of treatment condition (0.75 vs. 25 Hz) for SOL. Group (order of treatment arm) and day of treatment were not significant factors and no significant interactions between factors were identified for any of the variables tested. Comparison of each treatment condition to baseline indicated that treatment with 0.75 Hz SDR-tES significantly reduced sleep onset latency (SOL), and increased: time asleep and sleep efficiency (SE). Treatment with 25 Hz also significantly reduced SOL and increased SE compared to baseline. A multiple comparisons contrast run within the repeated measures ANOVA, revealed a limited interaction for SOL between treatment order and treatment condition for the group that received 0.75 Hz first (*p* < 0.025).

**Table 2:** Sleep characteristics comparing 0.75 Hz stimulation with same subject baselines and 25 Hz active control.

	Baseline	0.75 Hz	*p* (0.75 vs. Baseline)	25 Hz	*p* (25 vs. Baseline)	Treatment-dependent effect
SOL (min)	74.2 (33.2)	35.9 (23.5)	**1.5 E−07**	52.3 (44.7)	**0.011**	*F* (1,22) = 7.07; *p* = 0.014
WASO (min)	48.9 (23.2)	49.0 (19.3)	n.s.	44.5 (17.3)	n.s.	n.s.
Time Asleep (min)	378.9 (58.2)	402.7 (60.0)	**0.013**	380.3 (57.4)	n.s.	n.s.
SE (%)	75.7 (6.7)	82.6 (4.7)	**1.8 E−06**	80.5 (9.1)	**0.018**	n.s.

Values are mean +/− (SD). Significant *p*-values are bolded. n.s., not significant. SOL, sleep onset latency, WASO, wake after sleep onset, SE, sleep efficiency.

**Figure 3:**
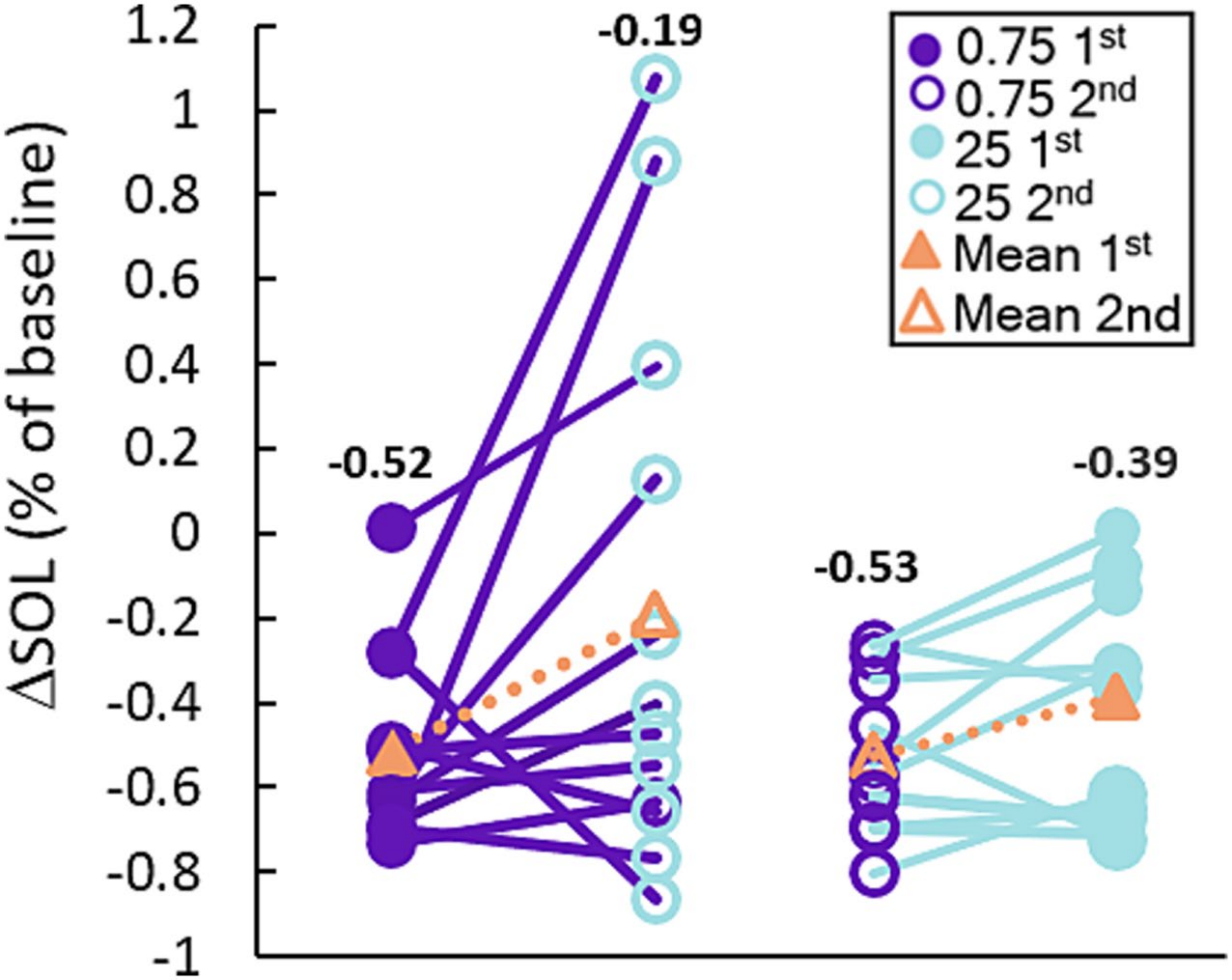
0.75 Hz stimulation improves SOL more consistently than 25 Hz. The plot shows the response of each participant to each treatment arm organized based on the order in which they were received. Treatment with 0.75 Hz consistently improves SOL regardless of whether it was given first or second, but treatment with 25 Hz shows a weaker effect when given second. The mean reduction for each group is shown with the orange triangles and the corresponding number above each set of points.

**Figure 4:**
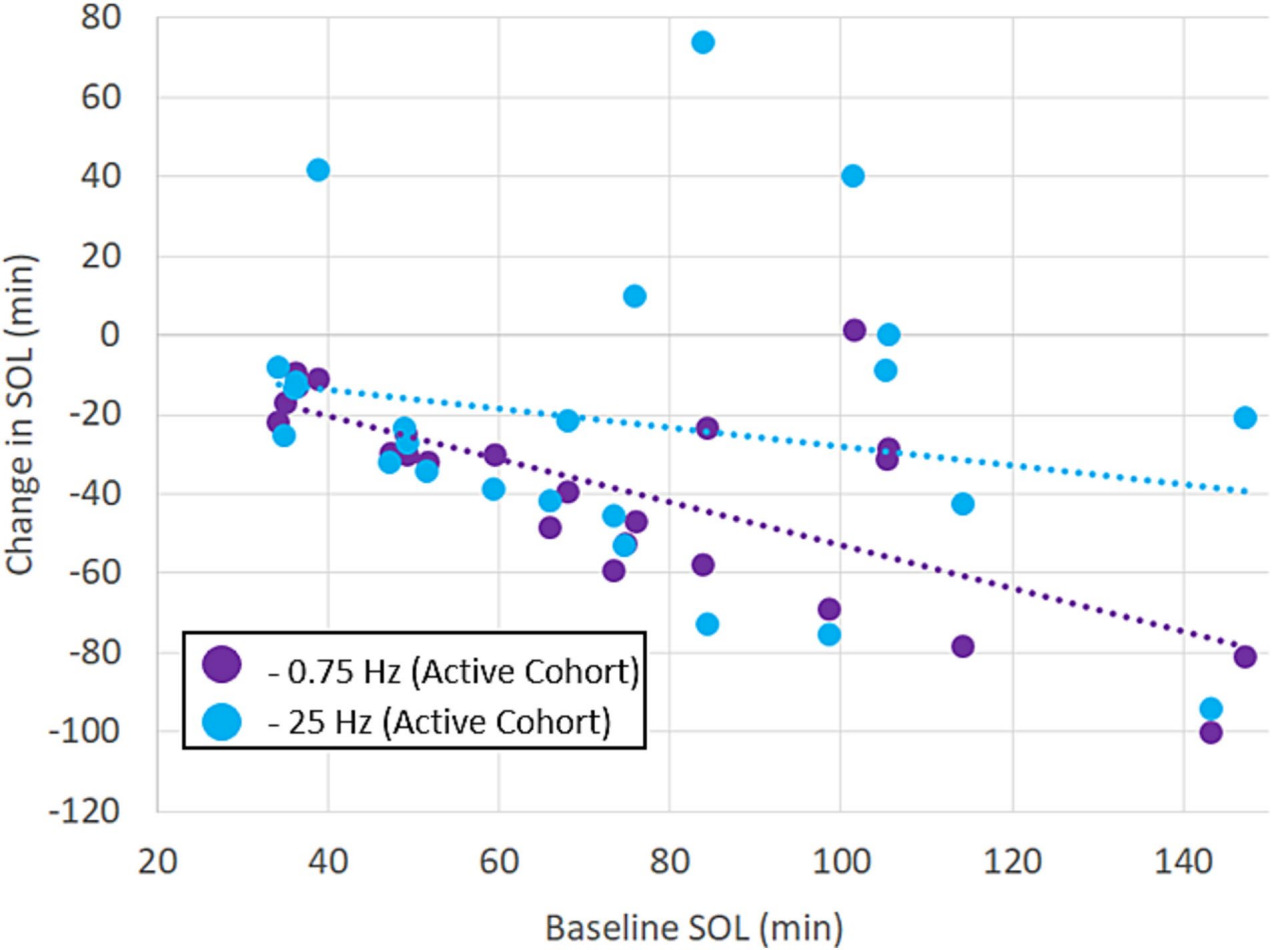
Benefits of treatment with 0.75 Hz are proportional to untreated baselines. The scatterplot shows the results of treatment with 0.75 Hz tES and 25 Hz tES relative to each participant’s baseline. The linear trendlines are fit to each treatment condition. The response to 0.75 Hz treatment is strongly correlated (Pearson’s *r* = −0.71, *p* = 1.1E−4). Correlation was not significant with 25 Hz stimulation (*r* = −0.21, *p* = 0.33).

Figure 3 shows the breakdown of participants’ SOL according to treatment order. The plots show that the average reduction in SOL with 0.75 Hz was >50% and was consistently observed regardless of treatment order. 23 out of 24 participants realized a reduction in SOL with 0.75 Hz treatment. By comparison, 25 Hz stimulation *increased* SOL in 5 out of 24 participants and showed order-dependent differences in the cohort means with larger reductions in SOL observed when it was given first. While 25 Hz strongly reduced SOL in certain participants, only 0.75 Hz treatment consistently and significantly reduced SOL regardless of treatment order. A closer look at SOL reveals a strong correlation between baseline and the response to treatment. Figure 4 shows the average change in SOL for each participant in response to 0.75 Hz treatment and our 25 Hz active control as a function of their pre-treated baseline. The linear trendlines describe a strong and significant correlation with 0.75 Hz treatment (Pearson’s *r* = −0.71, *p* = 1.1E-4), but no correlation with 25 Hz treatment (*r* = −0.21, *p* = 0.33).

The smaller improvements in sleep outcomes observed with 25 Hz stimulation suggest either a less effective response to higher frequency treatment, and/or some amount of placebo effect. The possibility of placebo was investigated in a separate cohort of 23 participants, comparing the efficacy of 0.75 Hz SDR-tES with a limited stimulus sham on summary sleep behaviors (SOL, TST, SE). The results indicated a similarly strong response to 0.75 Hz compared to sham stimulation (see Supplementary materials) suggesting placebo from wearing the device in the absence of prolonged stimulation is negligible.

### 3.2 Subjective measures of insomnia and anxiety

**Figure 5:**
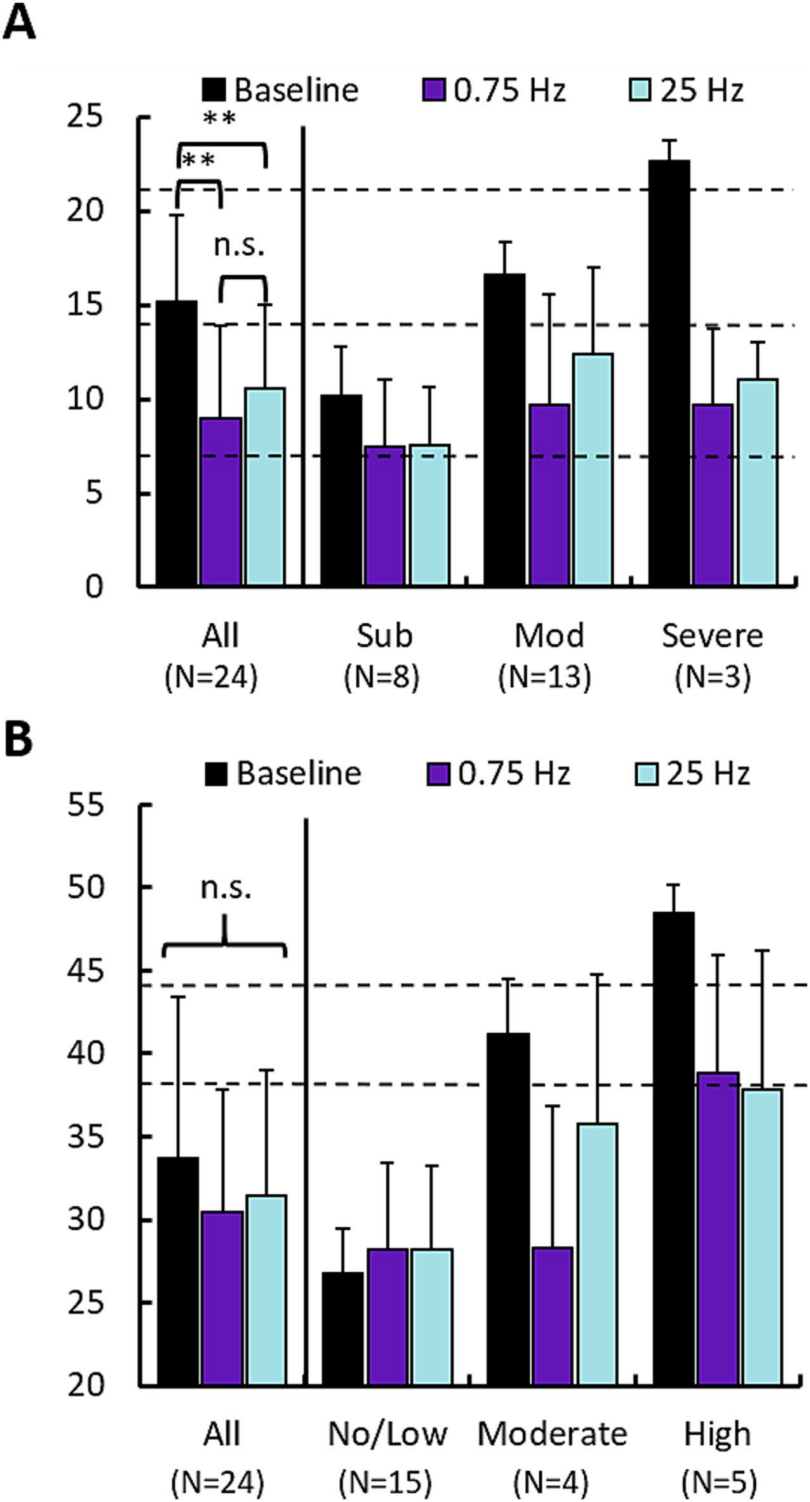
0.75 Hz and 25 Hz stimulation improve subjective measures of insomnia and anxiety. **(A)** Changes in Insomnia Severity Index (ISI) scores for 0.75 Hz and 25 Hz treatment arms compared with baseline. Left set of bars shows the mean of “All” ISI scores across the cohort (Baseline = 15.2 ± 4.6, 0.75 Hz = 9.0 ± 4.9, 25 Hz = 10.5 ± 4.4). Paired *t*-tests showed that both 0.75 Hz stimulation (*p* = 1.3E-5) and 25 Hz stimulation (*p* = 3E-5), led to significant reduction of ISI scores. Differences between 0.75 Hz stimulation and 25 Hz stimulation were not significant. The right 3 sets of bars shows scores broken out according to categorical levels observed during baseline. Dashed lines indicate cutoffs in scores for each category: “Subthreshold” 8–14 (*N* = 8), “Moderate” 15–21 (*N* = 13), “Severe” 22–28 (*N* = 3). **(B)** Changes in State Trait Anxiety Inventory scores for 0.75 Hz and 25 Hz treatment arms compared with baseline. No significant differences were identified between group means. Dashed lines indicate cutoffs in scores for each category: “No/Low” 20–37 (*N* = 15), “Moderate” 38–44 (*N* = 3), “Severe” 45–80 (*N* = 6). In both panels **(A,B)**, error bars are standard deviation; *p < 0.05, **p < 0.01. Statistics computed only across scores for the entire cohort (“All”). No significant differences were observed between 0.75 Hz and 25 Hz treatment.

We used the Insomnia Severity Index (ISI) and the State Trait Anxiety Index (STAI, State sub form) to survey participants’ perceptions of their insomnia and anxiety symptoms, respectively. Figure 5 shows mean responses across participants following baseline, and both treatment arms across the cohort and also segregates participants according to the categorical label of their baseline scores (e.g., “Subthreshold”). Statistics were computed only for the cohort averages at the left of each plot. Figure 5A describes the change in ISI scores. Both 0.75 Hz and 25 Hz treatment significantly reduced perceived insomnia symptoms. Results of an ANOVA test on all participants indicated a significant effect of treatment condition (*F* = 10.32, *p* < 0.005) but no effect of treatment order. Paired *t*-tests between conditions revealed significant difference between each treatment condition and baseline, but no significant differences in ISI scores between 0.75 Hz and 25 Hz treatment. Those with baselines described as “Moderate” or “Severe” had their scores reduced to “Subthreshold” levels. Those participants with baseline levels of “Subthreshold” insomnia still displayed reduced scores, but remained “Subthreshold.”

Figure 5B describes the change in STAI scores. The prevalence of anxiety was lower compared to insomnia with *N* = 9 participants being categorized as having “Moderate” or “Severe” anxiety vs. 15 participants that showed “No/Low” anxiety. Results of the ANOVA test indicated no significance across the cohort for either treatment condition or order. Similar to the ISI, both treatment arms provided nominal relief to those with elevated anxiety scores. Both 0.75 Hz and 25 Hz treatment reduced anxiety in participants with baseline scores in the “Moderate” and “Severe” groups, in both cases reducing scores by a full categorical level.

Only one participant in our study scored sufficiently high on the CES-D to indicate the possibility of depression, and therefore the data are not presented in the figure. We report the data here for transparency. Among the 23 participants with “No depression” mean ± standard deviation scores were 11.0 ± 3.2, 11.1 ± 5.3, and 10.9 ± 4.2 for the baseline, 0.75 Hz, and 25 Hz conditions, respectively. In the one participant demonstrating “Moderate depression” scores were 24, 8, and 17 for the baseline, 0.75 Hz and 25 Hz conditions, respectively.

### 3.3 Increases in slow wave power are uncorrelated with SOL changes

**Figure 6:**
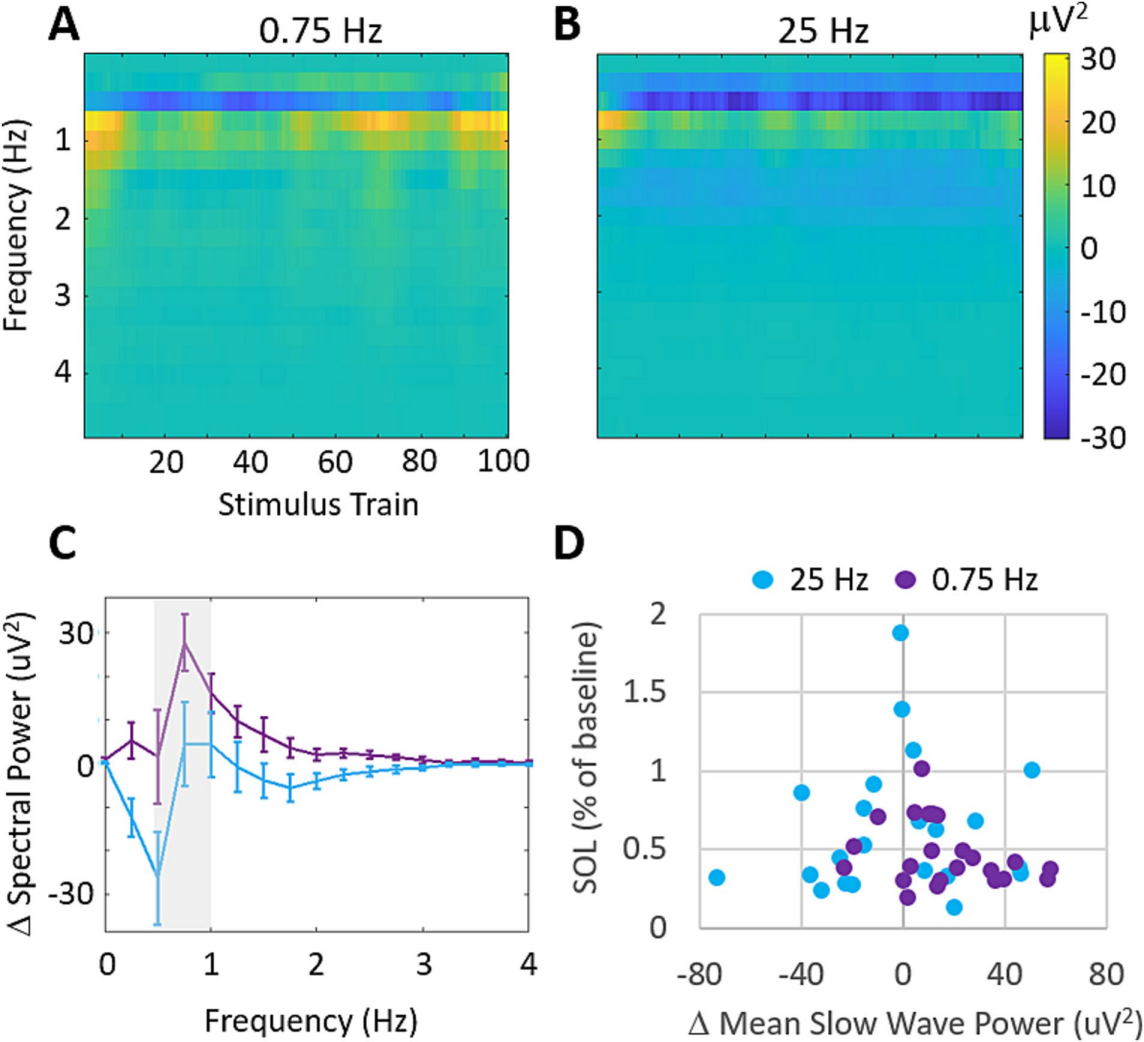
Stimulation evokes increases in slow wave spectral power that are uncorrelated with SOL. **(A)** Mean changes in spectral power evoked by each train of 0.75 Hz stimulation. Increases are observed around the stimulation frequency of 0.5–1 Hz. Frequencies higher than 4 Hz showed average differences <2mV^2^ and are not shown. **(B)** Mean changes in spectral power evoked by each train of 25 Hz stimulation. Increases are observed around 1 Hz but are comparably weaker than 0.75 Hz stimulation. **(C)** Mean changes in delta band (0–4 Hz) spectral power over the final 10 stimulation cycles for each condition. Error bars represent SEM. **(D)** Changes in SOL as a function of changes in mean slow wave power over the final 10 stimulation cycles. (Pearson’s correlation across both treatment groups *r* = −0.11, *p* = 0.45).

Using the three frontal EEG channels of the wearable device, we investigated the stimulation evoked changes in spectral power during treatment with 0.75 Hz and 25 Hz. Figure 6 shows the across participants mean changes in spectral power relative to a 60s baseline collected before stimulation. Figure 6A shows that 0.75 Hz stimulation leads to increases in spectral power around the stimulation frequency which are strongest at the start and end of stimulation. By comparison, 25 Hz produces very small increases in spectral power which are mostly apparent at the start of stimulation (Figure 6B). Frequencies above 4 Hz did not demonstrate any significant changes in spectral power from baseline and are not shown. Figure 6C shows the mean change in spectral power across slow wave frequencies (>0.5–1 Hz) in response to the final 10 stimulation trains where stimulation evoked difference between conditions were largest. While 0.75 Hz shows stronger increases in spectral power than 25 Hz around the stimulation frequency (gray shaded bar), these differences are not significant (*p* = 0.16). Figure 6D shows the relationship between changes in slow wave power averaged in response to the final 10 stimulation trains and the changes in SOL for each participant. A weak, and insignificant correlation is observed for all data (0.75 Hz and 25 Hz; Pearson’s *r* = −0.11, *p* = 0.45).

### 3.4 Increases in delta band coherence are modestly correlated with SOL changes

**Figure 7:**
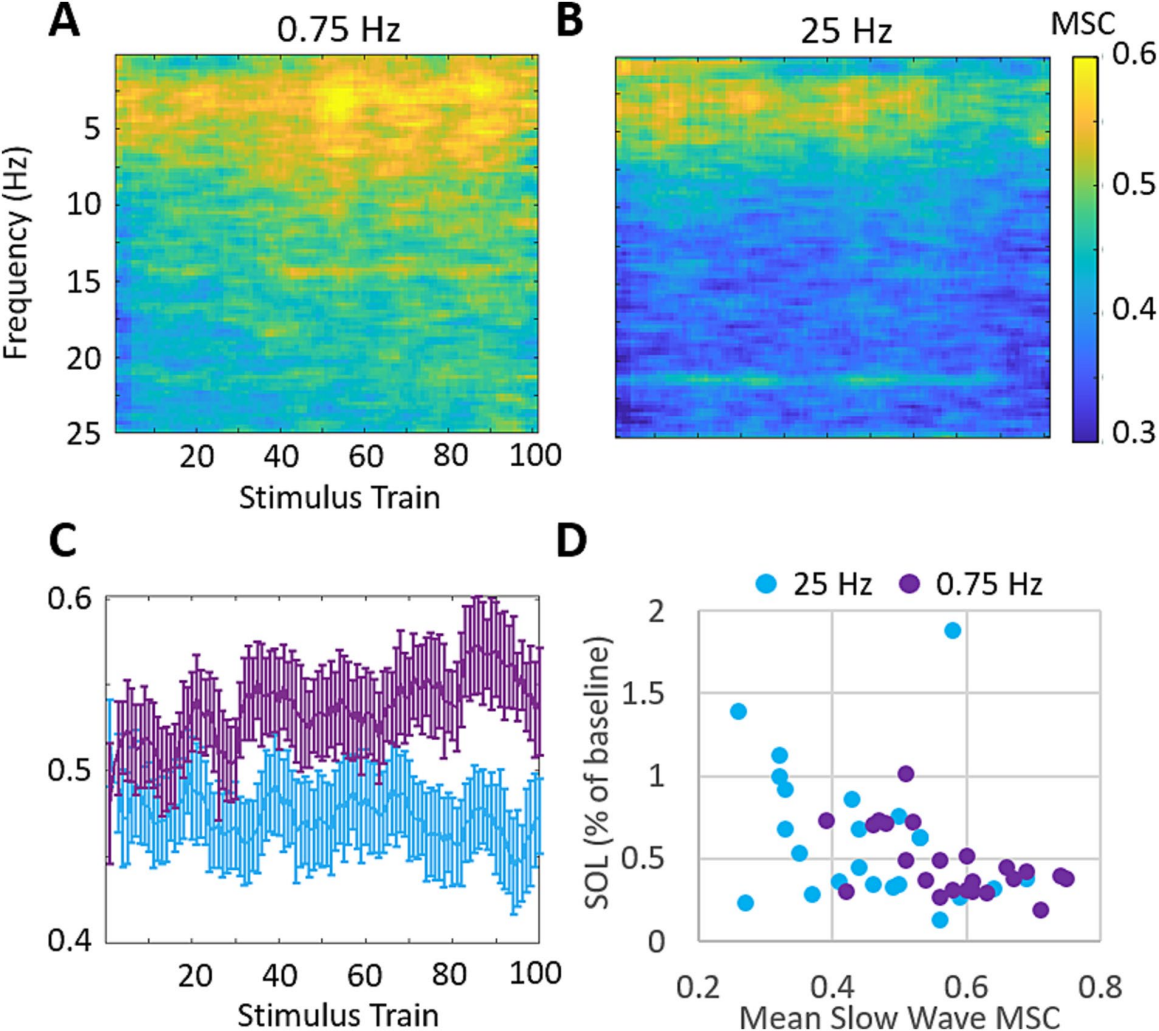
0.75 Hz stimulation evokes increases in magnitude squared coherence (MSC) that are correlated with SOL. **(A)** Mean MSC following each train of 0.75 Hz stimulation. MSC increases are observed across the delta band (0–4 Hz) and get progressively stronger with more stimulation. Mean MSC at frequencies above 25 Hz showed no relevant increases in response to either treatment and are not shown. **(B)** Mean MSC following each train of 25 Hz stimulation. 25 Hz stimulation does not similarly increase MSC over time. **(C)** Mean MSC in response to each stimulation train over delta band frequencies between 0 and 4 Hz for each condition. Error bars represent SEM. **(D)** Changes in SOL as a function of MSC across the delta band over the final 20 stimulation cycles. (Pearson’s correlation across both treatment groups *r* = −0.44, *p* = 0.002).

We also investigated the stimulation evoked changes in magnitude squared coherence (MSC) between the left and right EEG channels. Figure 7 shows the across participants mean MSC following each stimulation train. 0.75 Hz treatment leads to increasing MSC in the delta band (0–4 Hz) across the time of stimulation. 25 Hz treatment does not demonstrate similar increases in MSC during the time of stimulation and shows lower coherence overall (Figures 7A,B). Figure 7C shows the mean MSC across the delta band in response to each stimulation train. 0.75 Hz treatment shows a modest and gradual increase over the time of stimulation which peaks after ~80 stimulation trains. 25 Hz treatment shows little to no change over the time of stimulation. The mean difference in MSC between treatment conditions in response to the final 20 stimulation trains is significant (ΔMSC = 0.12, *p* = 9E−5). Figure 7D shows the relationship between mean slow wave MSC over the final 20 stimulation trains and the changes in SOL for each participant. A moderate and significant correlation is observed (Pearson’s *r* = −0.44, *p* = 0.002) suggesting that left/right slow wave coherence is a stronger predictor of changes in SOL than slow wave spectral power in our cohort.

### 3.5 Changes in SOL are not stimulation amplitude-dependent

**Figure 8:**
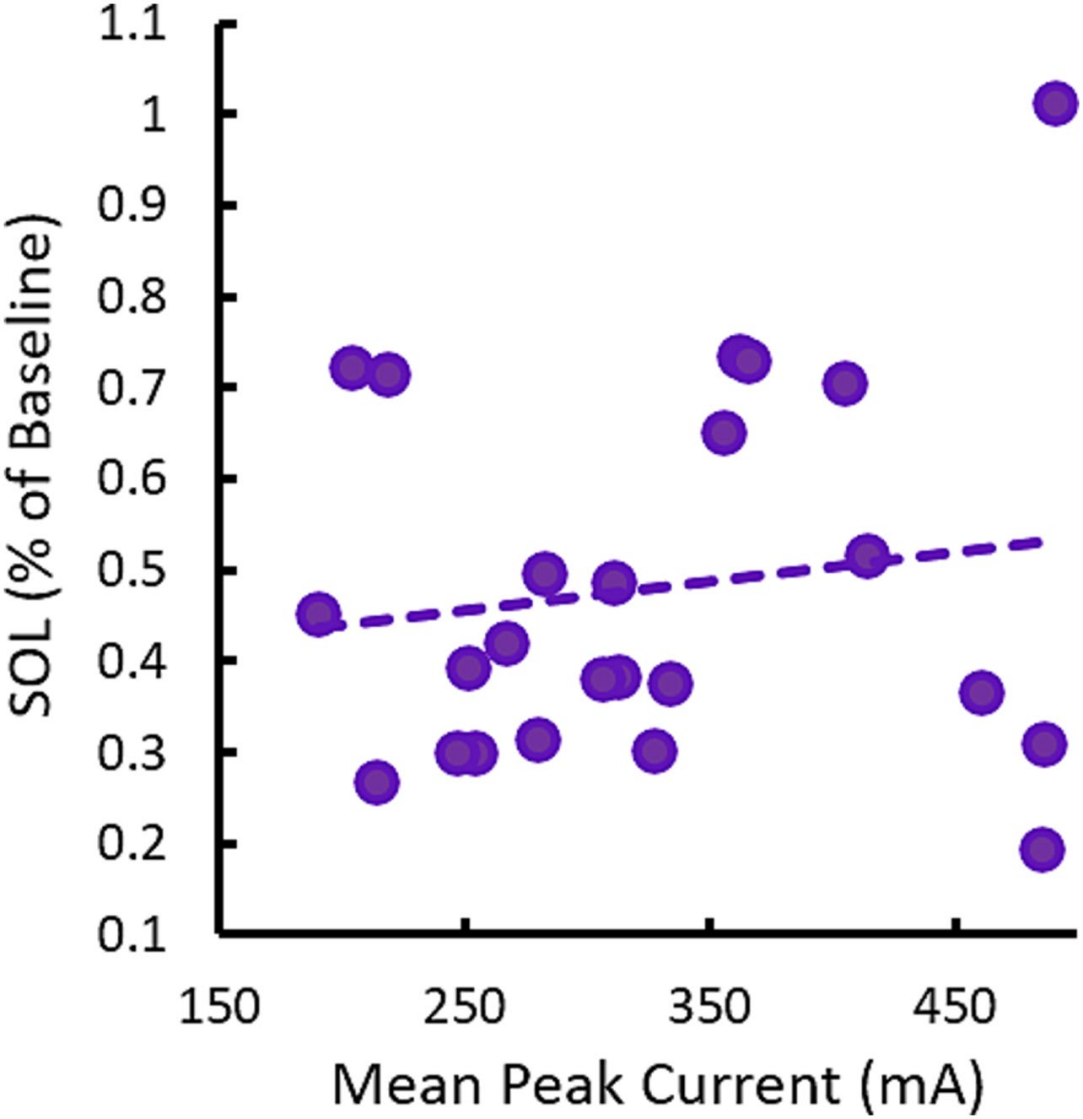
Changes in SOL in response to 0.75 Hz treatment are not dose-dependent. The scatterplot shows mean (across nights) changes in SOL as a function of the mean peak current (across stimulation trains and nights). Each point represents a single participant. The trendline shows there is no correlation between SOL and dose, *R* = 0.15, *p* = 0.49.

0.75 Hz treatment used a variable current amplitude which ranged between 150 μA to 500 μA depending on the input impedance of the participants’ skin prior to each stimulation train. This led to each participant receiving a different dose of stimulation as characterized by mean peak current across the treatment session(s). Figure 8 describes SOL as a function of the average dose of 0.75 Hz stimulation in each participant. No correlation is observed. Indeed, some of the strongest responders in terms of reduced SOL received low doses of stimulation that were less than the mean dose of 326 μA and the single non-responsive participant received among the highest doses delivered in the study. The lack of dose-dependent response is also supported by results in our supplemental sham cohort (see Supplementary materials). In this cohort, 0.75 Hz stimulation was applied consistently at 260 μA, but displayed similarly strong reductions in SOL.

## 4 Discussion

In this study, we have demonstrated a translational technology for administering tES in the home that is effective at significantly reducing SOL in those suffering from insomnia. The technology is easy to use and was well tolerated across participants.

### 4.1 Comparison on SDR-tES efficacy

**Figure 9:**
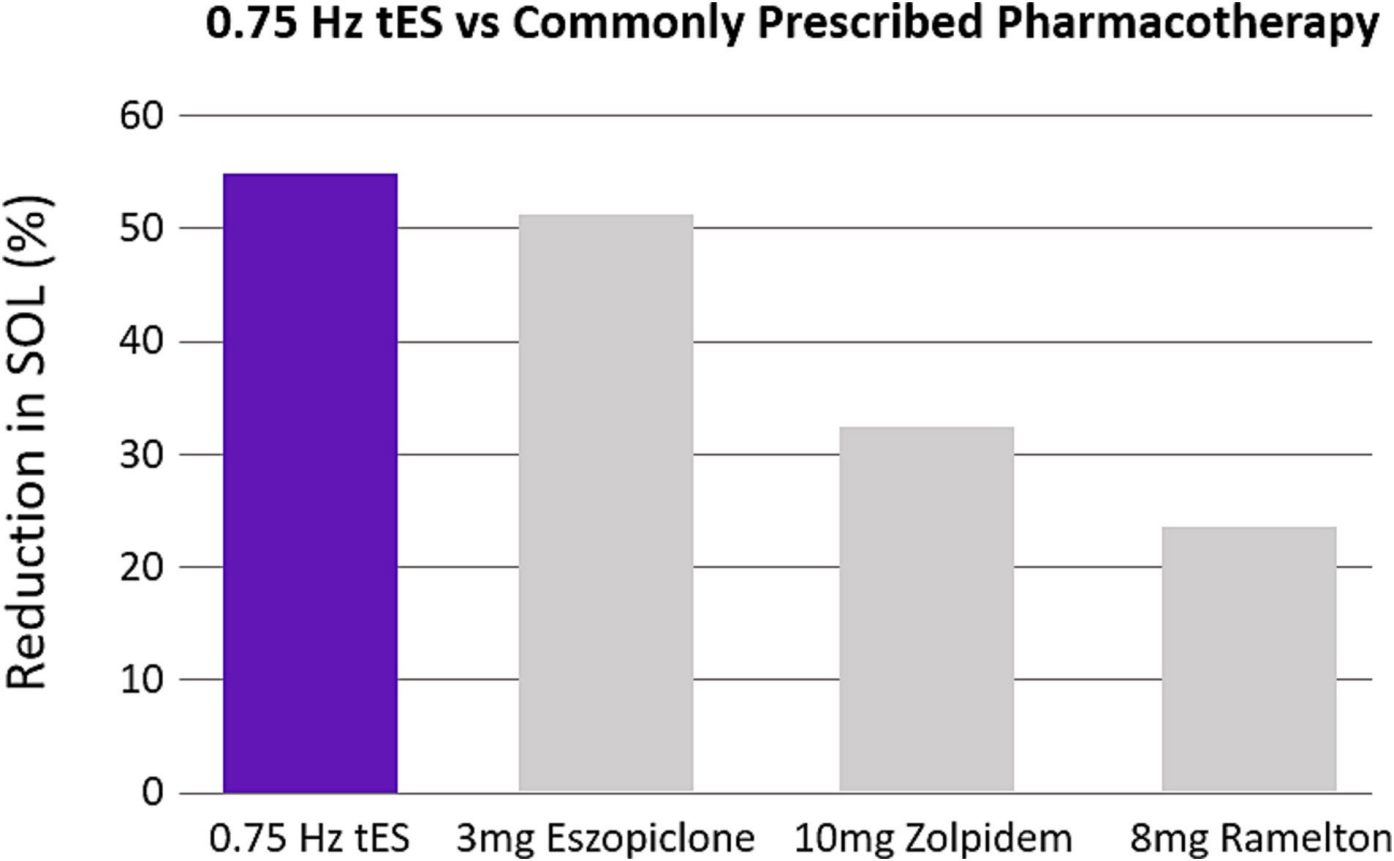
0.75 Hz treatment is more efficacious than pharmacotherapy. The bar plots were constructed from meta-analysis on clinical recommendations for pharmacotherapy (Sateia et al., 2017). The three most studied compounds are shown. Values are expressed as a percentage reduction compared to sham.

We demonstrated that application of 0.75 Hz SDR-tES for 30 min prior to bedtime dramatically reduced SOL. Treatment also increased time asleep and sleep efficiency and reduced the subjective symptoms of insomnia and comorbid anxiety relative to pre-treated baselines. Reductions in SOL were also significantly stronger with 0.75 Hz treatment compared to our active control using 25 Hz stimulation and a limited stimulation sham (see Supplementary materials). The magnitude of reduction in SOL and improvement in sleep efficiency observed here is similar to benefits observed with cognitive behavioral therapy (CBT) and stronger than the effects observed with the most commonly prescribed pharmacotherapies (Cellini et al., 2019; Research, 2013; Axelgaard, 2022; Thielscher et al., 2015). Figure 9 shows the reduction in SOL observed with our wearable device compared with the results of a meta-analysis of three of the most commonly prescribed and recommended compounds for clinically treated sleep onset insomnia (Sateia et al., 2017). We have focused on SOL as the primary application for this technology as it is the most common symptom among those that suffer from insomnia and presents a significant need (Bjorøy et al., 2020).

Conversely, the increases in time asleep observed with use of our wearable, are smaller than effects observed with those therapies, a result likely attributable to the limited duration of tES induced changes in excitability (Nitsche and Paulus, 2000; Nitsche and Paulus, 2001). In this study, we chose to have participants remove the wearable device prior to initiating sleep. However, there is significant research suggesting that neuromodulation during sleep may lead to larger improvements in sleep architecture (Cellini et al., 2019; Ladenbauer et al., 2017; Marshall et al., 2006) including in those with insomnia (Saebipour et al., 2015). These approaches could be easily combined with the wearable used in this study to potentially capture these additional benefits and improve outcomes for sleep maintenance insomnia.

### 4.2 Neural correlates of SOL improvements

Previous work using 0.75 Hz transcranial direct current stimulation has demonstrated significant increases in slow wave spectral power that were not observed in this study. The observation of only nominal increases in slow wave power could be due to the timing or duration of stimulation. We stimulated during periods of pre-sleep wakefulness, whereas previous studies have delivered stimulation during non-rapid eye movement sleep stage 2 and 3, when slow oscillatory activity is prevalent (Cellini et al., 2019; Ladenbauer et al., 2017; Marshall et al., 2006). Alternatively, it is possible that large increases were not observed due to inconsistencies in the test conditions. Spectral power is a signal amplitude-based measurement that is sensitive to parameters such as skin impedance that varied significantly during at-home use of our device. By comparison, cross channel coherence lacks this dependency and was significantly correlated with improvements in SOL in our study. Increases in coherence are consistent with a theorized and demonstrated mechanism of tES to improve functional connectivity in cortical networks around the stimulation frequency (Liu et al., 2018; Neuling et al., 2013).

### 4.3 Placebo vs. non-specific stimulation effects

In this study, we compared efficacy of our 30-min 0.75 Hz SDR-tES treatment to a 30-min 25 Hz active control. Our 25 Hz active control showed a more varied response across participants that was significantly different from pre-treated baselines depending on whether it was received first or second. The order-dependent differences suggest that at least part of the efficacy observed in response to the 25 Hz treatment could be due to placebo. This is supported by the consistent response to 0.75 Hz treatment regardless of order, and the higher variability in response to 25 Hz treatment overall.

Creation of an effective placebo control (sham) condition is difficult with tES because participants can feel the delivery of the electrical current. In our supplemental cohort, we found that 0.75 Hz treatment significantly reduced SOL compared to a sham (see Supplementary material). The similarity in SOL reduction suggests the SOL in participants receiving sham stimulation was not significantly different than pre-stimulation baselines. Thus, wearing the device in the absence of stimulation likely does not produce a strong placebo effect. This suggests that the smaller improvements in SOL observed in response to 25 Hz treatment could be due to a non-specific stimulation effect that is independent of stimulation frequency.

In response to other forms of treatment, meta-analysis has documented the strong tendency towards placebo effects in those being treated for insomnia. Winkler and Rief (2015) found that for those being treated with pharmacotherapy, placebo alone produced an effect size (Hedges *g*) of 0.35 for objective measurements of SOL and 0.31 for SE. This accounted for roughly 64% of the total effect of pharmacotherapy treatment. Similarly strong placebo effects have been reported in other meta-analyses for objective and subjective measurements of insomnia (Bélanger et al., 2007; Yeung et al., 2018) and across different types of treatment (Jiang et al., 2019; Jiang et al., 2019). We observed a significantly higher effect size on SOL with 0.75 Hz (*g* = 1.33) than 25 Hz treatment (*g* = 0.56). If one assumes that placebo alone may result in an effect size of *g* = 0.35 then roughly 63% of the observed effect with 25 Hz treatment is due to placebo and this number increases to 100% if considering only the group where 25 Hz was delivered in the second treatment arm (*g* = 0.34). By comparison, the effect size with 0.75 Hz treatment remains robust even after accounting for placebo regardless of whether it was delivered first (*g* = 1.44) or second (*g* = 1.25) where placebo could account for 24 and 28% of the total effect size, respectively.

Similar to other studies, we observed a strong improvement in subjective perceptions of insomnia and anxiety symptoms with treatment (Yeung et al., 2018). Our observed improvements in the ISI and STAI in both treatment conditions are not consistent with the observed differences across objective measures of sleep obtained with actigraphy, and suggest that these self-report measures are, unsurprisingly, more susceptible to placebo effects.

### 4.4 Use of neuromodulation in insomnia

A large number of research studies and a smaller number of commercially available devices have applied neuromodulation with the goal of improving insomnia outcomes and met with limited success. A recent meta-analysis of studies using neuromodulation for treatment of insomnia found that tES did not significantly change objective measures of sleep, while repetitive transcranial magnetic stimulation (rTMS) showed significant improvements in SE, TST, SOL, and WASO (Ma et al., 2021). Both tES and rTMS showed significant improvements in subjective measures of sleep quality consistent with this study. Across rTMS studies, the weighted mean difference for SOL was −9.78 min which is a smaller difference than the −38 min reported in our study. Changes in TST (37.25 vs. 24 min) and WASO (−27.86 min vs. no change) were larger for rTMS than those reported here, while changes in SE (7.91 vs. 6.85) were similar. This would suggest potentially larger efficacy for sleep maintenance insomnia from rTMS. However, rTMS is currently applied in the clinic and may not easily be translated to home use. Furthermore, as previously noted, impacts of tES can be possibly extended to improve sleep maintenance through closed-loop application during sleep (Saebipour et al., 2015).

Despite limited efficacy data, tES is easily applied and has been developed for use in multiple wearable technologies for sleep. For example, cranial electrotherapy stimulation (CES) delivers electrical current to the ear lobes and has been investigated in multiple studies but has not been shown to improve objective sleep measures (Lande and Gragnani, 2013; Wagenseil et al., 2018). More recently, a wearable headband which applies transcranial alternating current stimulation (tACS) across the sagittal midline of the forehead at theta and alpha (5–11 Hz) frequencies was shown to improve sleep duration but failed to report any improvement in SOL relative to a sham (Ayanampudi et al., 2023). Another similar device using tACS delivered at 77.5 Hz was used in a larger scale clinical trial but only reported subjective outcome measures which, as previously discussed and shown here, may not correlate with objective improvements in sleep quality (Wang et al., 2020).

The stronger efficacy demonstrated in this study is likely due to significant differences in the stimulation approach. First, our stimulating electrode montage applies oscillating anodal direct current bilaterally to the frontal cortex. Our results demonstrate that left/right synchronization around the stimulation frequency of 0.75 Hz is correlated with improvement in SOL. Bilateral transcranial direct current stimulation (tDCS) stimulates both hemispheres simultaneously and is more likely to produce this synchronization compared to tACS applied across the sagittal midline which produces alternating depolarizing and hyperpolarizing inputs across the hemispheres.

The specific use of 0.75 Hz direct current stimulation has a long history of application *during sleep* to improve memory and promote slow wave activity in the brain (Cellini et al., 2019; Ladenbauer et al., 2017; Marshall et al., 2006), but has not been previously investigated as a pre-sleep intervention for insomnia. This frequency differs significantly from other tES trials which have applied frequencies ranging from 5 to 1,000 Hz (Lande and Gragnani, 2013; Wagenseil et al., 2018; Ayanampudi et al., 2023; Wang et al., 2020; Shekelle et al., 2018; Zhou et al., 2020). Here, and previously, we varied this paradigm by delivering short, repetitive, stimulation trains (SDR-tES) enabling more frequent interrogation of the brain’s response during stimulation (Cellini et al., 2019). Another possibility is that these shorter periods of stimulation may be more effective than the standard approach of continuous stimulation over many minutes. For example, attractor models of neural dynamics have suggested that continuous application of tES pulses may be suboptimal by attempting to entrain brain activity to unnatural states (Lisitsyn and Ernst, 2019). According to this model, the preferred approach is to use minimal perturbation to gently push the network into a desired state. Future studies will need to investigate the parameters of stimulation train length and intertrain intervals to determine whether any comparative benefits exist from shorter vs. continuous application of tES.

### 4.5 Limitations

There are several limitations to the current study. We chose to monitor sleep using wrist worn actigraphy rather than the gold standard of polysomnography (PSG). The FitBit Inspire 3 used in this study enables easy measurement of sleep behaviors in the home and may reduce confounds associated with poor sleep in the lab (Edinger et al., 1997), however actigraphy is known to be less accurate than PSG in all regards (Chinoy et al., 2022; Lim et al., 2023). Meta-analysis has shown that sleep onset is consistently underestimated by actigraphy and sleep tracking wearables including the FitBit Inspire 2 used in this study (Chinoy et al., 2022; Scott et al., 2020). However, the reductions in SOL observed with treatment from stimulation (−38 min and −22 min for 0.75 Hz and 25 Hz respectively), significantly exceed these reported inaccuracies which are −4 min on average (Chinoy et al., 2022; Scott et al., 2020).

Use of our wearable device at home also leads to potential inconsistencies given the inability to monitor participants’ behavior during and around use. This is potentially problematic for consistent collection of EEG signals that may encounter significant noise when the headband is not correctly positioned or during undesirable behaviors (e.g., movement, teeth clenching). These limitations were viewed as a favorable tradeoff for observing the impact of our stimulation paradigm during ecologically valid test conditions in the home. However, characterization of whole brain signals in response to treatment and precise measurement of sleep architecture will require future studies with PSG.

### 4.6 Future clinical application

We’ve demonstrated a patient-ready wearable prototype device which can be easily applied to clinical practice. tES has been determined to be extremely safe when delivered within the bounds of our stimulation parameters used here (Scott et al., 2020). It carries an extremely favorable side effect profile to pharmacotherapy and the benefits to SOL are observed after just one night’s use. The correlation between changes in SOL and left/right frontal coherence suggest that closed-loop delivery with this measure as the target state could yield stronger or quicker results and should be explored. Taken together, our results provide evidence for an exciting new therapeutic paradigm for treating insomnia and the future of neuromodulation in insomnia research.

## Data Availability

The raw data supporting the conclusions of this article will be made available by the authors, without undue reservation.
