# Supplementary material for: A randomized study on the effect of a wearable device using 0.75 Hz transcranial electrical stimulation on sleep onset insomnia
Source: Front Neurosci. 2024 Oct 23;18:1427462. doi: 10.3389/fnins.2024.1427462 (PMC11537953; doi:10.3389/fnins.2024.1427462)
Supplement: Supplementary file 1 [file Data_Sheet_1.docx]

Supplementary Materials and Methods

Sham Study Design & Stimulation Protocol

In a pilot study, we compared the efficacy of 30 minutes of treatment with 260μA, 0.75 Hz SDR-tES to a limited-stimulus sham using the same tES parameters but delivered for only 90 seconds. The two conditions were delivered using an earlier prototype of our wearable device used in the active control cohort described in the main manuscript. The 90 second sham was intended to control for placebo arising from the sensation of stimulation. The sensation from stimulation with our device typically wanes after 2-3 minutes. 90 seconds of stimulation using our SDR-tES paradigm was hypothesized to be so small that it would be unlikely to evoke meaningful neural effects (note this translates to only 40 seconds of actual stimulation time). The stimulation waveform and protocol were otherwise identical to our active control cohort except for the amplitude which was fixed at 260μA.

Sleep behaviors between the sham and treatment condition were compared in a population of otherwise healthy adults with self-reported symptoms of sleep onset insomnia. Inclusion criteria for the study required subjects to be between 21-70 years old. Subjects were screened for issues with sleep onset using the Pittsburgh Sleep Quality Index Component 2 sub form (score>=3). Exclusion criteria were identical to our active control cohort described in the main manuscript.

Our study protocol is shown in Figure S1. Each participant spent two weeks enrolled in the study and made three visits to our laboratory. We used the Philips Actiwatch 2 to track the sleep of participants at home. The Actiwatch does not provide data on sleep stages but was a widely used tool in research for identifying sleep onset and duration prior to it being discontinued in 2022. During each week, participants were sent home with a Teledyne sleep wearable device (headset and accompanying mobile phone) and asked to wear the device for 4 consecutive nights of the week. Each participant received both the treatment and sham protocols over sequential weeks with stimulation occurring on the same days of the week (e.g. Monday-Thursday). Participants were block randomized for order of each study arm and balanced for gender. Study population demographics for the pilot study are shown in Table S1.

We enrolled 31 participants over the course of the pilot study with 23 completing the study. 7 were dismissed due to insufficient number of completed nights of treatment in at least one condition. 1 withdrew following treatment with 0.75 Hz stimulation due to discomfort. Data analysis and statistics were performed in the same way as our active control cohort described in the main manuscript.

Supplementary Results

The purpose of this cohort was to test for placebo from wearing the headset in the absence of consistent stimulation. Table S2 shows the results comparing 0.75 Hz stimulation to our sham for our primary sleep variables of SOL, time asleep and SE. Compared to the sham, 0.75 Hz treatment significantly reduces SOL and increases SE. Differences in time asleep were not significant. This is likely due to a ceiling effect in this cohort which had healthy sleep durations of greater than 7 hours even during sham treatment. The differences between sham stimulation and 0.75 Hz treatment are described in Figure S2. Similar to the results comparing 0.75 Hz stimulation to untreated baseline, the plot shows a strong negative correlation indicating that the benefits of stimulation are proportional to the deficits observed with sham stimulation (r = -0.9, p = 1.2E-8). The plot shows that all but one participant receiving the treatment demonstrated improvement in their SOL (>95% response rate).

Supplementary Figures & Tables

**Figure S1.** **Within-subjects, sham-control crossover design.** Our pilot study observed sleep at home using a wrist worn Philips Actiwatch 2. Our 0.75 Hz SDR-tES pre-sleep intervention was tested over 4 days using our wearable neurotechnology. Both treatment conditions (0.75 Hz treatment or sham) were tested in each subject and the order was counterbalanced across subjects. Each participant made three visits to our laboratory for data and equipment exchange. actigraphy data, was collected during lab visits according to the schedule shown and compared across treatment arms.

**Fig. S2. Changes in SOL with 0.75 Hz stimulation are proportional with SOL during sham stimulation.** The scatterplot shows the SOL recorded in the same participants in response to sham stimulation (x-axis) and the relative difference in response to 0.75 Hz stimulation (y-axis). N=23 participants are shown. The scatterplot is fit well by a linear trend with slope of -0.74.

**Table S1. Sham cohort participant population demographics.**

|  | 0.75 Hz 1^st^ | Sham 1^st^ |
| --- | --- | --- |
| *Gender* |  |  |
| Female | 9 | 8 |
| Male | 2 | 4 |
| *Age* |  |  |
| Median | 47 | 38 |
| Range | 28-67 | 22-61 |
| 21-32 | 3 | 2 |
| 33-45 | 1 | 7 |
| 46-58 | 5 | 2 |
| 59-70 | 2 | 1 |

**Table S2.** **Sleep characteristics comparing 0.75 Hz stimulation with limited stimulus sham.**

|  | Sham | 0.75 Hz | p |
| --- | --- | --- | --- |
| SOL (min) | 47.8 (30.4) | 19.9 (18.6) | **9.1E-5** |
| Time Asleep (min) | 439.5 (59.6) | 466.9 (84.2) | n.s. |
| SE (%) | 82.7 (5.1) | 88.9 (2.4) | **3.2E-5** |

Values are mean +/- (SD). Significant p-values are bolded. n.s. – not significant
