# Supplementary figures and images for: A randomized study on the effect of a wearable device using 0.75 Hz transcranial electrical stimulation on sleep onset insomnia
Source: Front Neurosci. 2024 Oct 23;18:1427462. doi: 10.3389/fnins.2024.1427462 (PMC11537953; doi:10.3389/fnins.2024.1427462)

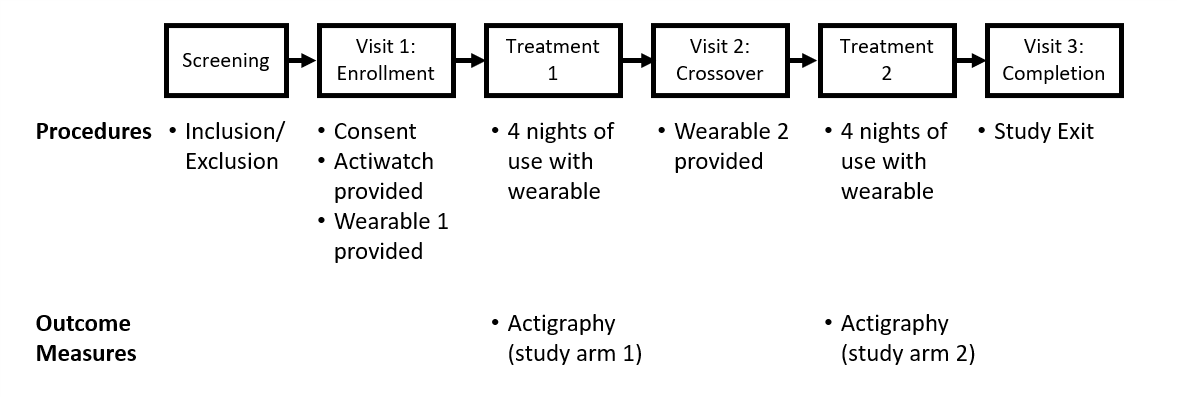

Supplement: Supplementary file 2 [file Image_1.tif]

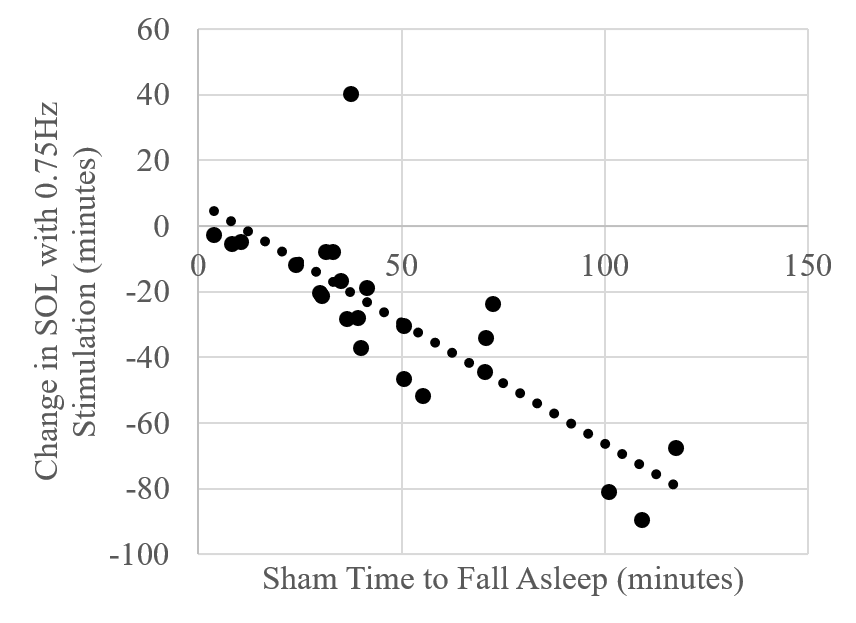

Supplement: Supplementary file 3 [file Image_2.tif]
